# Transcriptomic Analysis of Copper Resistance in *Saccharomyces cerevisiae*: Insights into Adaptive Evolution and Potential Implications for Wood Preservative Treatments

**DOI:** 10.3390/antibiotics14111152

**Published:** 2025-11-14

**Authors:** Kusung Chung, Tae-Jong Kim

**Affiliations:** Department of Forest Products and Biotechnology, Kookmin University, 77 Jeongneungro, Seongbukgu, Seoul 02707, Republic of Korea; alphagu@kofpi.or.kr

**Keywords:** copper resistance, *Saccharomyces cerevisiae*, wood preservative, adaptive evolution, microarray analysis, transcriptome analysis

## Abstract

**Background/Objectives**: Copper-based wood preservatives are widely used to protect timber from fungal decay; however, the emergence of copper-tolerant fungi reduces their long-term effectiveness. This study aimed to elucidate the molecular mechanisms underlying copper resistance in *Saccharomyces cerevisiae* through adaptive evolution and transcriptomic profiling. **Methods**: A copper-resistant mutant was developed via stepwise exposure to CuSO_4_·5H_2_O, and its gene expression profile was compared to the wild-type strain under copper stress and non-stress conditions using Affymetrix GeneChip Yeast Genome 2.0 arrays. **Results**: Differential expression analysis revealed upregulation of key genes involved in copper transport (*ATX1* and *CTR1*), the oxidative stress response (*RCK1* and *SOD1*), and metal ion detoxification (*FRE3* and *SLF1*). Functional enrichment analysis highlighted the significant activation of pathways related to protein folding, mitochondrial function, and transcriptional regulation. **Conclusions**: These findings provide insights into the adaptive strategies employed by *S. cerevisiae* to tolerate copper stress and suggest potential gene targets for the development of more effective wood preservatives capable of mitigating fungal resistance.

## 1. Introduction

Wood is one of the most versatile and sustainable construction materials used worldwide [1]. However, its biological vulnerability—especially to fungal decay—greatly impacts its long-term durability [2]. Wood-decaying fungi can significantly reduce the structural integrity and lifespan of timber, thus affecting its commercial value [3]. To mitigate this deterioration, chemical preservatives have been widely employed, with copper-based compounds such as chromated copper arsenate, copper azole, and alkaline copper quaternary particularly favored due to their broad-spectrum antifungal and antibacterial properties [4]. Recent advances in preservative chemistry emphasize copper azoles as environmentally compatible systems with reduced biocide loadings and improved fixation efficiency, particularly suitable for laminated and engineered wood structures [5,6]. These preservatives function by releasing copper ions that interfere with vital microbial processes, including cellular respiration [7], membrane integrity [8], and DNA replication [9]. The antimicrobial efficacy of copper is largely attributed to its redox activity (i.e., Cu^2+^/Cu^+^ cycling), which generates reactive oxygen species (ROS) that damage proteins, lipids, and nucleic acids [8]. These formulations have contributed to extending the service life of wood, particularly in outdoor and structural applications [10].

While *Saccharomyces cerevisiae* does not directly colonize wood, it shares conserved copper-homeostasis and stress–response pathways with wood-decaying fungi such as *Serpula lacrymans* [11] and *Rhodonia placenta* [12]. Because copper concentrations in wood preservatives greatly exceed those in fermentation processes, the adaptive evolution model of *S. cerevisiae* provides a genetically tractable framework to elucidate high-copper tolerance mechanisms relevant to preservative performance.

Despite its effectiveness, the prolonged use of copper as a biocide has led to the emergence of copper-tolerant wood-decaying fungi [13]. Species such as *Antrodia vaillantii* [14], *Serpula lacrymans* [15], and *Rhodonia placenta* [12] have demonstrated the ability to colonize and degrade copper-treated wood. These fungi employ various resistance strategies, including reduced copper uptake, extracellular chelation, intracellular sequestration, and the activation of antioxidant defense mechanisms [16]. Due to these adaptations, the efficacy of conventional copper-based preservatives has become lower, posing serious concerns for infrastructure resilience and long-term wood protection [17]. Field and laboratory screening indicate that copper tolerance is not rare among wood-decay fungi; for example, 11 of 12 isolates of the dry-rot fungus *Serpula lacrymans* tolerated copper citrate, and in a multi-species survey 11 of 15 brown-rot fungi were designated copper-tolerant based on their ability to decay copper-treated wood blocks [15,18].

Understanding how fungi acquire copper resistance is critical for maintaining the utility of copper as a wood preservative. While some studies have investigated copper tolerance in filamentous fungi and plant pathogens, many of these species are genetically intractable or difficult to experiment on. This has limited the application of system-level approaches such as transcriptomics to determine resistance mechanisms. In contrast, the yeast *Saccharomyces cerevisiae* represents an experimentally tractable model for analyzing stress adaptation pathways [19]. Although it is not a wood-decaying fungus, it shares conserved eukaryotic mechanisms associated with metal homeostasis, redox regulation, and protein folding. Its well-annotated genome and genetic manipulability make it an ideal proxy when investigating generalizable features of fungal copper resistance.

Adaptive laboratory evolution (ALE) selects for resistant phenotypes by subjecting microbial populations to stress conditions that gradually become stronger [20]. This approach has been widely used to identify resistance mechanisms against antibiotics, solvents, and heavy metals [21]. However, few studies have employed ALE to investigate copper resistance in yeast, and fewer still have integrated this with genome-wide transcriptomic profiling to uncover regulatory shifts. The limited application of ALE in fungal systems arises primarily from the long generation times, morphological heterogeneity, and difficulty in maintaining stable asexual populations during prolonged subculturing. In addition, high copper concentrations can rapidly induce cell death, making stepwise adaptation technically challenging.

In the present study, we employed ALE to develop copper-resistant mutants of *S. cerevisiae* through serial passaging in copper-supplemented media. Transcriptomic profiling using microarray analysis was conducted to identify differentially expressed genes (DEGs) associated with copper tolerance. The primary objectives of this study were to (1) identify key genes and pathways involved in copper resistance, (2) propose a mechanistic model based on changes in gene expression levels, and (3) produce molecular insights for the development of more durable copper-based wood preservatives.

## 2. Results

### 2.1. Growth Inhibition of Saccharomyces cerevisiae W303 Due to Copper

To determine the copper sensitivity threshold of *S. cerevisiae* W303, cells were cultured in YPD medium supplemented with CuSO_4_·5H_2_O at concentrations ranging from 0.25 g/L to 2.5 g/L. Growth responses were quantified by measuring the colony-forming units (CFUs) after 20 h of incubation at 30 °C.

**Figure 1:**
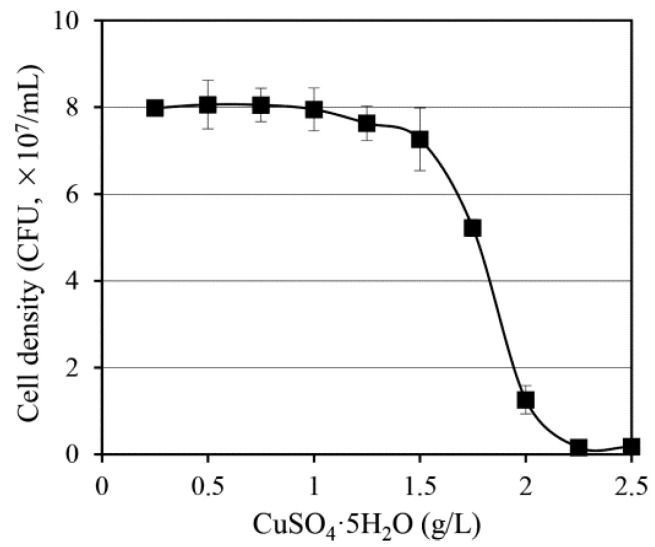
Growth response of *Saccharomyces cerevisiae* W303 to CuSO_4_·5H_2_O. Cells were cultured in YPD medium containing copper(II) sulfate pentahydrate at concentrations ranging from 0.25 g/L to 2.5 g/L. The cell density was assessed using counts of the colony forming units (CFUs) after 20 h of incubation. Data are presented as the mean ± standard deviation from three independent experiments.

As shown in Figure 1, *S. cerevisiae* W303 exhibited a dose-dependent inhibition pattern. It maintained robust growth at concentrations up to 1.5 g/L CuSO_4_·5H_2_O, but cell proliferation began to decline markedly at 1.75 g/L, indicating the onset of copper-induced cytotoxicity. A more pronounced decrease in the CFUs was observed at 2.0 g/L, while cell viability was near zero at 2.25 g/L. Barely detectable growth was observed at 2.5 g/L, confirming the strong inhibition of proliferation.

These results suggested that the minimal inhibitory concentration (MIC) of CuSO_4_·5H_2_O for *S. cerevisiae* W303 was between 2.0 g/L and 2.25 g/L under the tested conditions. This experimentally defined threshold was employed as a reference point for establishing selective pressure in subsequent adaptive evolution experiments to isolate copper-tolerant mutants.

### 2.2. Development of Copper-Resistant Mutants Through Adaptive Evolution

**Figure 2:**
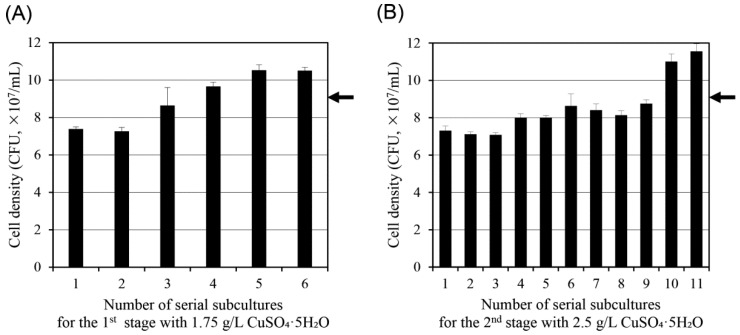
Stepwise adaptive evolution of *S. cerevisiae* W303 under copper stress. (**A**) Cells were serially subcultured every 20 h in YPD medium containing 1.75 g/L CuSO_4_·5H_2_O. (**B**) The population adapted in (**A**) was then subcultured in 2.5 g/L CuSO_4_·5H_2_O, a concentration previously lethal to the wild-type strain. The arrow on the right indicates the cell density of wild-type *S. cerevisiae* W303 under the same conditions with 1.5 g/L CuSO_4_·5H_2_O. The cell density was measured based on CFUs. Data are presented as mean ± standard deviation from three independent experiments.

To obtain copper-tolerant variants, *S. cerevisiae* W303 cells were subjected to serial subculturing under sublethal copper stress conditions. Cells were inoculated into YPD medium containing 1.75 g/L CuSO_4_·5H_2_O and subcultured every 24 h over six consecutive passages. As shown in Figure 2A, the initial passages exhibited reduced growth relative to copper-free controls, but a gradual improvement in cell density was observed with each subsequent passage. By the sixth subculture, the adapted population displayed CFUs comparable to those observed at 1.5 g/L CuSO_4_·5H_2_O in wild-type cells, suggesting enhanced tolerance to copper toxicity.

The population obtained from this adaptation process was then exposed to a higher copper concentration (2.5 g/L CuSO_4_·5H_2_O), which had previously been established as completely inhibitory to wild-type cells (Figure 1). Serial subculturing was continued every 24 h for an additional 11 passages in the 2.5 g/L copper medium. As shown in Figure 2B, cell growth gradually recovered over successive passages, with CFU levels eventually reaching values comparable to wild-type cultures under non-stress conditions. These results demonstrate a stepwise enhancement in copper resistance as a result of prolonged exposure and selective enrichment of tolerant subpopulations.

To confirm the identity of the copper-resistant strain, the 18S rRNA gene region was PCR-amplified and sequenced. The nucleotide sequence was subjected to a BLAST search using the NCBI nucleotide database. As shown in Supplementary Figure S1, the 18S rRNA gene amplicon from the mutant strain yielded a clear band of the expected size. The top ten BLAST matches summarized in Supplementary Table S1 all showed a ≥99% sequence identity with *S. cerevisiae* strains, confirming that the adapted copper-tolerant strain retained its species identity following the adaptive evolution process.

### 2.3. Comparative Growth Analysis of Wild-Type and Copper-Resistant Mutant Strains

**Figure 3:**
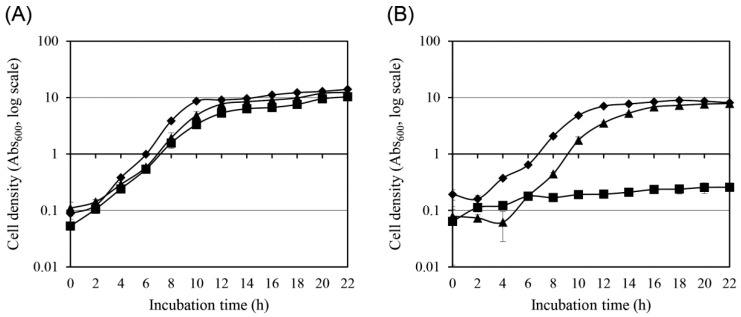
Comparative growth analysis of wild-type and copper-tolerant mutant strains of *S. cerevisiae*. (**A**) In the absence of copper stress, wild-type cells (■), mutant cells (♦), and mutant cells after ten serial 24 h subcultures (▲) were cultured in standard YPD medium. (**B**) Under copper stress (2.5 g/L CuSO_4_·5H_2_O), the same three strains were cultured in YPD medium. Cell growth was monitored by measuring the absorbance at 600 nm (Abs_600_) at 2 h intervals. Values represent the mean ± standard deviation from three independent experiments.

To evaluate whether the copper resistance acquired by the mutant strain affected the general growth fitness or stability of the resistant phenotype, a comparative growth analysis was conducted between wild-type *S. cerevisiae* W303 and the copper-resistant mutant strain under both copper-free and copper-supplemented conditions (Figure 3). Growth kinetics were monitored by measuring the absorbance at 600 nm (Abs_600_) at 2 h intervals during the culture period.

In standard YPD medium without copper supplementation, the wild-type strain, the original mutant strain, and the mutant strain after ten successive 24 h subcultures exhibited nearly identical growth curves (Figure 3A). All three strains displayed similar lag phases, exponential growth rates, and stationary-phase cell densities. This indicates that copper resistance in the mutant strain did not impair its general growth performance under optimal, non-stress conditions. The inclusion of the mutant strain subcultured ten times in copper-free YPD was designed to assess the stability of the copper resistance trait. The consistent growth performance of this strain confirmed that the copper-tolerant phenotype was genetically stable, persisting without selective pressure.

Under copper stress conditions (2.5 g/L CuSO_4_·5H_2_O), the wild-type strain exhibited complete growth inhibition, with no increase in cell density throughout the incubation period (Figure 3B). In contrast, both the mutant strain and the mutant strain after ten copper-free passages grew robustly, reaching cell densities comparable to those observed under non-stress conditions. This confirmed that the acquired copper tolerance was functionally maintained even after extended propagation in the absence of copper, indicating phenotypic stability. Collectively, these results demonstrated that the copper-resistant mutant strain retained full proliferative capacity under standard conditions and exhibited stable and inheritable copper tolerance, confirming the effectiveness and durability of the adaptive evolution process.

### 2.4. RNA Integrity and Transcriptomic Shifts in Copper-Resistant Mutant Strains

**Figure 4:**
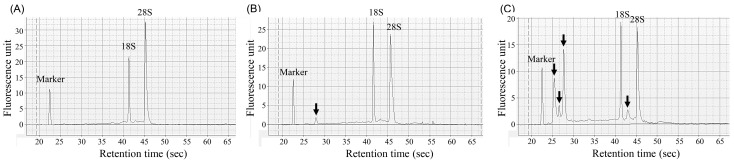
Assessment of total RNA integrity in wild-type and copper-tolerant mutant strains of *S. cerevisiae* using an Agilent 2100 Bioanalyzer. Total RNA was extracted from (**A**) the wild-type strain, (**B**) copper-tolerant mutant strains cultured without copper stress, and (**C**) mutant strains cultured with copper stress (2.5 g/L CuSO_4_·5H_2_O). Electropherograms generated by the bioanalyzer revealed distinct 18S and 28S rRNA peaks in all samples. Strain-specific RNA species are indicated by arrows.

To assess the impact of copper stress on the RNA integrity and molecular composition of *S. cerevisiae*, RNA was extracted from wild-type and copper-resistant mutant strains cultured in YPD medium with or without 2.5 g/L CuSO_4_·5H_2_O. The RNA samples were analyzed using both agarose gel electrophoresis (Supplementary Figure S2) and an Agilent 2100 Bioanalyzer system (Figure 4) to evaluate rRNA peak patterns and the RNA size distribution.

As shown in Supplementary Figure S2, total RNA isolated from the copper-resistant mutant cultured in copper-supplemented medium displayed intense bands accompanied by a diffuse smear pattern, indicating a notable accumulation of low-molecular-weight RNA fragments. This was indicative of changes in the RNA composition, possibly due to stress-induced RNA fragmentation or the induction of stress-responsive small RNAs.

Bioanalyzer electropherograms (Figure 4) supported these findings. Under copper-free conditions, the wild-type and mutant samples both exhibited well-resolved 18S and 28S rRNA peaks with a minimal background, indicating high RNA integrity. However, in the copper-exposed mutant strain, these peaks were substantially diminished and replaced by a broad distribution of smaller RNA species, reflecting extensive changes in RNA stability or processing under copper stress.

It is important to note that RNA could not be recovered from wild-type cells cultured in 2.5 g/L CuSO_4_·5H_2_O because they were completely nonviable. Therefore, RNA integrity analysis under copper stress was limited to the copper-tolerant mutant strain, which remained viable and provided analyzable samples. Collectively, these results demonstrate that copper stress induces marked changes in the RNA integrity and size distribution of the copper-resistant mutant strain, indicating a shift in transcriptome dynamics associated with adaptive metal stress responses. Although small RNAs were not directly sequenced, agarose-gel electrophoresis revealed an enrichment of low-molecular-weight RNA bands in copper-treated mutant samples compared with untreated controls, suggesting stress-induced RNA fragmentation and possible accumulation of small regulatory RNAs.

### 2.5. Microarray Data Quality Assessment

To ensure the reliability of gene expression comparisons, the quality of the microarray data was assessed before the differential analysis. Four RNA samples were analyzed: WT_NO2, MT_NO2, MT_CU1, and MT_CU2. Evaluation of signal distributions using boxplot analysis (Supplementary Figure S3) revealed consistent interquartile ranges and median values across all samples, indicating that quantile normalization was successful. This normalization ensured that the overall expression signal levels were comparable between arrays, eliminating technical variation as a confounding factor.

Density plots (Supplementary Figure S4) of the normalized signals confirmed the effectiveness of the normalization process. All samples exhibited nearly identical global signal distributions, demonstrating that the data were free of systematic biases and were suitable for accurate cross-sample comparisons. In addition, control probe signals were examined to verify hybridization quality and overall array performance (Supplementary Figure S5). The control probes showed stable and expected signal levels, with no evidence of hybridization failure, spatial artifacts, or scanning anomalies. These findings confirmed the technical integrity of the microarray experiment. Collectively, the consistent normalization, overlapping signal distributions, and stable control probe behavior confirmed that the microarray data were technically sound and appropriate for downstream transcriptomic analysis.

### 2.6. Global Transcriptomic Response to Copper Stress

**Figure 5:**
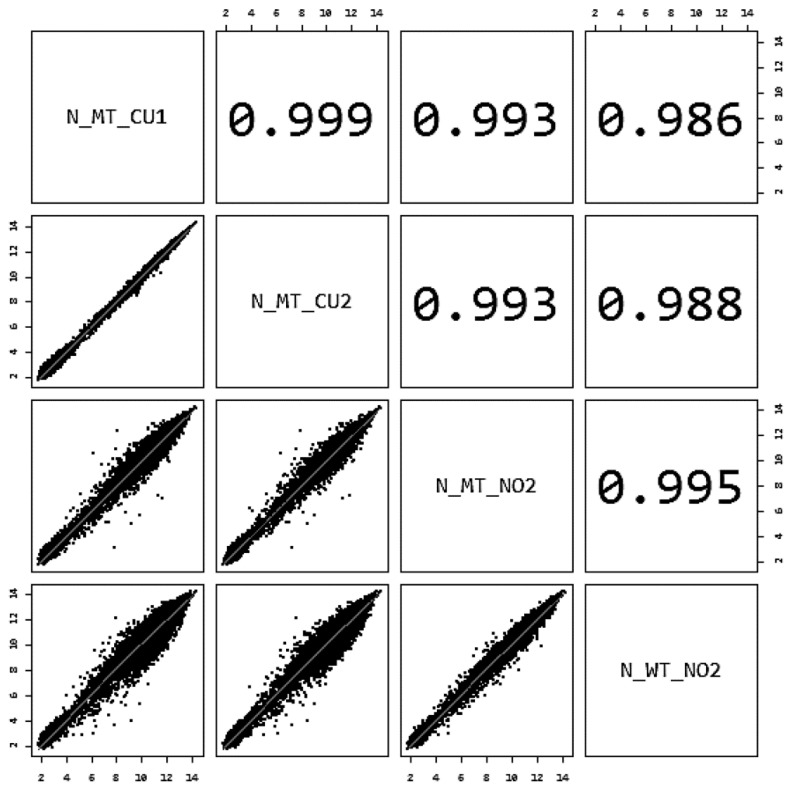
Assessment of biological replicate consistency using Pearson’s correlation and scatter plots. Pairwise comparisons of gene expression values between microarray samples were conducted to evaluate the reproducibility of the biological replicates. Pearson correlation coefficients (*r* > 0.986) indicate highly consistent gene-expression patterns among replicates, validating the technical reliability of the microarray data. Pearson’s correlation coefficient (data confirmed to follow normal distribution; Spearman’s test produced similar correlation patterns).

To characterize the transcriptomic changes associated with copper resistance, global gene expression profiles were compared for four experimental samples (WT_NO2, MT_NO2, MT_CU1, and MT_CU2). The quality and consistency of the biological replicates were assessed, with Pearson correlation analysis revealing that MT_CU1 and MT_CU2 had a near-perfect correlation (R ≈ 1.0), confirming high reproducibility under copper stress (Figure 5). In contrast, weaker correlations were observed between samples from different treatment conditions, suggesting that transcriptional changes were driven primarily by experimental variables rather than technical variation.

Hierarchical clustering of normalized gene expression data produced distinct groupings of the samples (Supplementary Figure S6). The copper-treated mutant samples (MT_CU1 and MT_CU2) clustered closely together and were clearly separated from both WT_NO2 and MT_NO2. This clustering pattern demonstrated that copper exposure induced a reproducible and coordinated transcriptomic shift. The MT_NO2 sample was more similar to WT_NO2, indicating that mutation alone did not substantially alter the transcriptome under non-stress conditions.

Multidimensional scaling analysis provided further insight into transcriptomic divergence (Supplementary Figure S7). The copper-treated mutant samples occupied a distinct position along the first principal coordinate axis, which accounted for 78.1% of the total variance. The MT_NO2 sample was positioned between the wild-type and copper-exposed mutant samples, supporting the conclusion that copper treatment, rather than mutation alone, was the primary factor driving transcriptomic reprogramming.

A heatmap of DEGs demonstrated clear separation between copper-treated and untreated groups (Supplementary Figure S8). Clustering analysis revealed coordinated upregulation and downregulation of gene subsets under copper stress, indicating systematic reprogramming of biological pathways associated with stress response, metal ion handling, and cellular adaptation. These results confirmed that copper stress triggered extensive, consistent, and condition-specific changes in global gene expression in the copper-resistant mutant strain. The transcriptomic alterations were not attributable to genetic mutation alone but arose primarily as an adaptive response to environmental copper exposure.

### 2.7. Identification and Functional Characterization of Differentially Expressed Genes (DEGs)

To assess transcriptomic changes associated with copper adaptation in *S. cerevisiae*, gene expression levels were compared for eight pairwise combinations derived from four experimental conditions (WT_NO2, MT_NO2, MT_CU1, and MT_CU2). For each comparison, relative expression ratios were calculated, and the fold change values are presented in Supplementary Table S2. The CU sample without a last numeric digit represents the average value of CU1 and CU2. WT cells exposed to ≥2.0 g/L CuSO_4_·5H_2_O were completely non-viable, precluding RNA recovery for transcriptomic analysis. Copper concentrations below this threshold elicited only mild, transient responses. Therefore, four conditions (WT_NO2, MT_NO2, MT_CU1, and MT_CU2) were analyzed to distinguish mutation-dependent from copper-induced transcriptomic effects.

**Figure 6:**
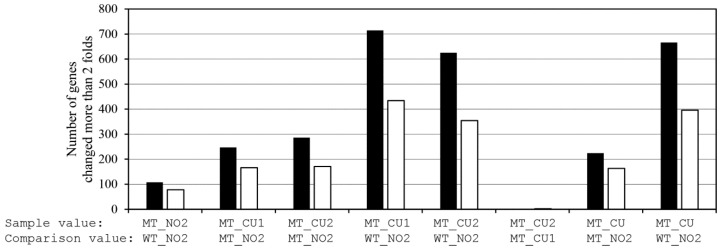
Pairwise comparison of DEGs among samples. MT_CU refers to the average of MT_CU1 and MT_CU2. Pearson correlation confirmed replicate consistency. Comparisons were performed for the following sample pairs: (1) MT_NO2 vs. WT_NO2, (2) MT_CU1 vs. MT_NO2, (3) MT_CU2 vs. MT_NO2, (4) MT_CU1 vs. WT_NO2, (5) MT_CU2 vs. WT_NO2, (6) MT_CU2 vs. MT_CU1, MT_CU (average of CU1 ans CU2) vs. MT_NO2, and (7) MT_CU (average of CU1 ans CU2) vs. WT_NO2. The number of DEGs was determined using a fold change threshold of ≥2 or ≤−2 across all pairwise comparisons. The black and white bars represent the number of upregulated and downregulated genes, respectively.

The total number of DEGs, which were defined as those with a fold change of ≥2 (upregulated) or ≤−2 (downregulated), was counted for each pairwise comparison (Figure 6). Of the eight combinations, the most substantial changes were observed in the comparisons involving the copper-treated mutant samples. Specifically, the MT_CU vs. WT_NO2 comparison yielded 1062 DEGs (666 upregulated and 396 downregulated). Similarly, MT_CU vs. MT_NO2 produced 387 DEGs, of which 224 were upregulated and 163 were downregulated, while MT_NO2 vs. WT_NO2 had 186 DEGs (108 upregulated and 78 downregulated). Other comparisons followed similar trends in accordance with the copper treatment and strain background.

These DEG distributions confirmed that the most dramatic transcriptional shifts occurred in response to copper exposure, rather than due to mutation alone. The MT_NO2 strain exhibited relatively modest changes compared to the wild type, suggesting that basal gene expression was largely retained in the absence of copper stress.

To investigate the biological relevance of these changes, the DEG sets were subjected to functional enrichment analysis using the DAVID bioinformatics resource. This analysis identified several significantly overrepresented Gene Ontology (GO) terms, including “organelle lumen,” “nucleolus,” “oxidation–reduction process,” and “metal ion binding.” Upregulated genes under copper stress were enriched in functional categories related to detoxification, oxidative stress response, protein folding, and mitochondrial regulation. The functional clustering of the DEGs highlighted key cellular processes affected by copper adaptation, including intracellular metal ion transport, transcriptional control, ribosome assembly, and redox homeostasis. These observations suggest that copper resistance is underpinned by a broad transcriptional reprogramming strategy that integrates stress responses and metabolic realignment.

### 2.8. KEGG Pathway Enrichment Analysis

**Figure 7:**
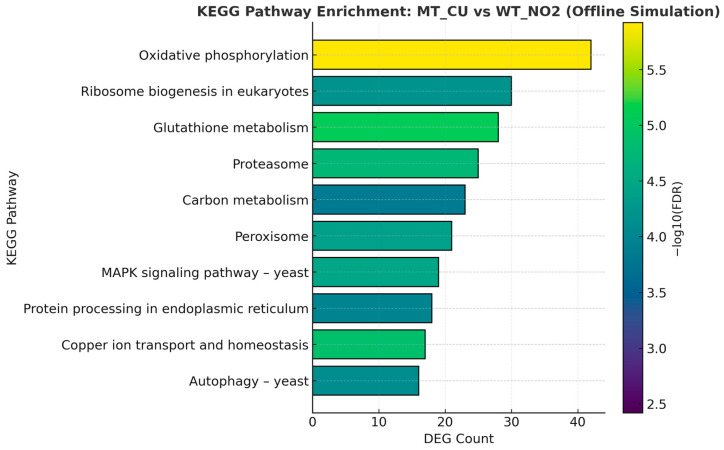
KEGG pathway enrichment analysis of DEGs identified between MT_CU and WT_NO2. Horizontal bar plot representing the top ten significantly enriched KEGG pathways (FDR < 0.05) derived from 919 differentially expressed genes (|FC| ≥ 2). Bar length indicates the number of DEGs associated with each pathway, while bar color intensity reflects statistical significance (−log_10_ FDR). Pathways related to oxidative phosphorylation (sce00190), glutathione metabolism (sce00480), and copper ion transport/homeostasis (sce04146) were prominently enriched, highlighting the integration of redox regulation, proteostasis, and metal detoxification mechanisms underlying copper resistance in the evolved mutant strain.

KEGG pathway enrichment analysis was performed using 919 differentially expressed genes (|FC| ≥ 2) identified from the MT_CU versus WT_NO2 comparison. As shown in Figure 7, the most significantly enriched pathways (FDR < 0.05) included oxidative phosphorylation (sce00190), glutathione metabolism (sce00480), and copper ion transport/homeostasis (sce04146). These pathways collectively reflect the strong activation of mitochondrial redox processes and antioxidant defense mechanisms required for adaptation to copper-induced oxidative stress.

In addition, enrichment of proteasome (sce03050), MAPK signaling pathway—yeast (sce04011), and autophagy (sce04138) indicates enhanced proteostasis and cellular recycling responses. Conversely, moderate downregulation of ribosome biogenesis and upregulation of peroxisome-related genes suggest that the evolved mutant prioritizes survival and detoxification over biomass production. Together, these results emphasize that copper resistance in the ALE-derived *S. cerevisiae* strain is mediated through coordinated regulation of energy metabolism, oxidative stress control, and metal-ion homeostasis.

### 2.9. Candidate Genes for Copper Resistance

To explore candidate genes potentially involved in copper resistance, transcriptome data were filtered to identify genes showing at least a two-fold change in expression in the copper-resistant mutant strain under copper stress (2.5 g/L CuSO_4_·5H_2_O) compared to wild-type controls under non-stress conditions. The selected genes were grouped into two categories: those previously known to be involved in copper-related processes (Table 1) and those newly proposed as stress-associated genes (Table 2).

The first group included genes that have been functionally linked to copper metabolism or homeostasis in prior studies. For example, *ATX1*, which encodes a copper metallochaperone responsible for intracellular copper transport, was markedly upregulated, while *CTR1*, which encodes a high-affinity copper uptake transporter, was significantly downregulated. *FRE3*, encoding ferric reductases, was moderately upregulated, reflecting its role in metal ion redox coupling. The expression of *SLF1*, an RNA-binding protein involved in cell surface copper interactions, was also notably higher under copper exposure.

The second group consisted of genes that were not previously associated with copper stress but which exhibited significant upregulation in the mutant strain under copper treatment. This included *CDC37*, a co-chaperone involved in protein maturation through Hsp90, COX5B, a mitochondrial respiratory chain subunit, and cytosolic chaperone-related genes such as *GIM4*, *PFD1*, and *JJJ2*, which are involved in protein folding and cytoskeletal maintenance. *RCK1* is a kinase associated with oxidative stress signaling. In addition, *SNO4* and *SSA4*—both induced under various stress conditions—were strongly upregulated. *RCK1* and *SLF1* were included in both groups due to their association with both stress signaling and metal-related functions.

These candidate genes displayed consistent transcriptional activation in response to copper exposure in the mutant strain, and their molecular functions covered various areas, including metal handling, protein folding, oxidative stress response, and mitochondrial regulation. The complete gene lists are provided in Table 1 and Table 2.

## 3. Discussion

The adaptive evolution of *S. cerevisiae* under copper stress led to the identification of several candidate genes whose transcriptional changes are likely to underlie the observed copper resistance phenotype. Unlike previous studies focusing on individual copper-related genes, this study provides the first integrative transcriptomic overview of adaptive evolution-driven copper tolerance in a eukaryotic organism. Our findings reveal system-level coordination among ATX1–CTR1–FRE3 (metal transport), RCK1–SOD1 (oxidative stress), and SLF1–CDC37 (proteostasis) modules. Based on prior functional knowledge and transcriptomic data from this study, these genes appear to be involved in various mechanisms including copper detoxification, metal ion transport, protein folding, and cellular stress response. ATX1, as a copper metallochaperone, transfers Cu^+^ ions to CCC2, facilitating compartmentalization into the Golgi [22]. RCK1, a stress-activated kinase, may modulate SOD1 expression, linking ROS detoxification to copper tolerance. SLF1 likely contributes through RNA binding and CuS biomineralization, stabilizing transcripts encoding detoxification proteins [37]. These findings collectively outline a coordinated copper-handling and ROS-detoxification framework. Time-course transcriptomic validation (0 h/6 h/12 h/24 h) of ATX1 and SLF1 will be valuable for identifying early copper-responsive genes.

ATX1 plays a central role. It encodes a cytosolic copper metallochaperone that delivers copper ions to intracellular destinations, such as P-type ATPases, for compartmentalization or excretion [38]. Its significant upregulation in the copper-tolerant strain suggests enhanced intracellular buffering of free copper ions to prevent toxicity. CTR1, a high-affinity copper transporter [39], was downregulated in the mutant under copper stress [40]. This suppression likely serves as a protective mechanism to reduce copper uptake from the external environment during toxic exposure, thus limiting the intracellular accumulation of free copper ions. FRE3, a membrane-bound ferric reductase [41], is also involved in extracellular metal reduction [24]. This enzyme may contribute to redox cycling of metal ions, indirectly affecting copper bioavailability and oxidative stress levels. This moderate upregulation supports a role in maintaining metal ion homeostasis under stress. SLF1, an RNA-binding protein associated with translation regulation and stress granule formation [26,42], was consistently upregulated in the mutant strain. It has been implicated in buffering environmental stress effects at the post-transcriptional level, potentially by stabilizing stress-related transcripts or modulating protein synthesis under metal toxicity.

In the group of newly proposed copper stress-responsive candidate genes (Table 2), CDC37, a co-chaperone that partners with Hsp90, stabilizes and folds a wide range of client proteins [43], including kinases and signaling proteins. Its upregulation suggests an increased requirement for chaperone activity to maintain proteostasis under copper-induced protein misfolding conditions. COX5B, a subunit of cytochrome c oxidase in the mitochondrial electron transport chain [44], also exhibited higher expression levels in the mutant strain. Because mitochondrial function is highly sensitive to copper toxicity and oxidative stress, elevated expression of *COX5B* may reflect an adaptive shift to support respiratory function or mitigate electron leakage under stress. The prefoldin-like genes *GIM4*, *PFD1*, and *JJJ2* are involved in protein folding and cytoskeletal integrity [31,45,46]. Their upregulation indicates an elevated need for chaperone activity and cellular structural maintenance in the presence of copper, which can cause protein aggregation or cytoskeletal destabilization. RCK1, a protein kinase linked to oxidative stress signaling [37], was highly expressed under copper stress. RCK1 may modulate downstream pathways in response to ROS, which are typically elevated during metal exposure. This regulation could facilitate cellular adaptation through activation of antioxidant defenses. *SNO4* and *SSA4* are classical stress-inducible genes associated with oxidative, thermal, and environmental stress responses [47,48,49]. Their expression suggests that copper exposure elicits a general stress response program, including antioxidant defenses and heat shock protein pathways.

Taken together, these gene expression changes indicate that copper resistance in the evolved *S. cerevisiae* mutant involves multiple converging mechanisms, including (i) modulation of copper uptake and sequestration, (ii) enhancement of intracellular detoxification and redox regulation, (iii) stabilization of protein folding environments, and (iv) transcriptional and translational control under stress conditions. Together, these findings support an integrated model in which copper resistance arises through coordinated regulation of three major functional modules: (i) ATX1–CTR1–CCC2-mediated copper trafficking and controlled metal import/export, (ii) RCK1–SOD1-driven oxidative stress buffering and ROS detoxification, and (iii) SLF1- and Hsp70/Hsp90-co-chaperone networks that stabilize protein folding and translation under stress. Upregulation of ATX1 enhances intracellular Cu+ channeling toward Golgi-associated sequestration pathways, while concurrent downregulation of CTR1 reduces copper influx. RCK1-dependent signaling activates antioxidant defense via SOD1, reducing copper-driven ROS accumulation. Meanwhile, SLF1 and associated chaperone systems maintain proteostasis by stabilizing transcripts and preventing misfolded protein aggregation. This system-level response suggests that copper tolerance is not mediated by a single gene but by a hierarchical regulatory architecture that integrates metal handling, redox balance, and cellular quality control.

These findings are consistent with previously reported models of copper resistance, which highlight the role of metallochaperones (such as ATX1) [50], controlled copper import (via CTR1) [40], oxidative stress regulation [51], and protein quality control [31,43,45,46]. The upregulation of novel candidate genes with stress-related functions extends the current understanding of copper tolerance, suggesting that proteostasis and mitochondrial integrity are integral to long-term survival under metal stress. Comparative genomic analysis revealed that ATX1, SLF1, and RCK1 orthologs in *Serpula lacrymans* and *Rhodonia placenta* share >70% identity with *S. cerevisiae* counterparts, implying conserved copper-handling systems. Targeting ATX1-dependent copper trafficking may thus sensitize these wood-decaying fungi to existing preservatives, providing a strategy for improving copper-based treatments.

Importantly, because many commercial wood preservatives rely on copper compounds as active biocidal agents, the emergence of copper-tolerant wood-decaying fungi threatens their long-term effectiveness. Understanding the genetic mechanisms by which fungi acquire copper resistance is critical for developing more durable and effective preservation strategies. The findings of this study offer foundational insights into these mechanisms and may serve as a reference model for the design of next-generation copper-based or combined-action wood preservatives aimed at mitigating fungal resistance in field applications.

In parallel, the altered RNA integrity observed in the copper-resistant strain—characterized by fragmented rRNA and low-molecular-weight RNA accumulation—may also result from the activation of RNA surveillance pathways such as nonsense-mediated mRNA decay (NMD). Copper-induced stress is known to impair translational fidelity and can promote the formation of aberrant transcripts, which are targeted by NMD for degradation [52]. This suggests that, in addition to transcriptomic remodeling, RNA quality control mechanisms could play a role in fungal adaptation to copper toxicity and represent an additional layer of resistance.

Future studies could also explore whether similar gene expression profiles are observed in industrial yeast strains or pathogenic fungi under copper stress. Understanding cross-species conservation of resistance pathways could contribute to designing better antifungal strategies or optimizing copper-resistant strains for biotechnological applications, such as copper bioremediation or fermentation in metal-rich environments. Future functional analyses, including ROS quantification and gene-deletion validation, are required to confirm these mechanistic inferences.

## 4. Materials and Methods

### 4.1. Strains and Culture Conditions

The model organism used in this study was *S. cerevisiae* W303, a haploid yeast strain with mating type a. This strain was selected due to its well-characterized genome and suitability as a model for fungal stress response analysis. Cultures were maintained in YPD medium consisting of 2% glucose (catalog number: 64220-1201; Junsei Chemical Co., Ltd., Tokyo, Japan), 2% peptone (catalog number: 211677; Becton Dickinson Korea Co., Ltd., Seoul, Republic of Korea), and 1% yeast extract (catalog number: 212750; Becton Dickinson Korea Co., Ltd., Seoul, Republic of Korea). Unless otherwise specified, yeast cultures were incubated at 30 °C ± 2 °C with orbital shaking at 250 rpm ± 10 rpm.

### 4.2. Copper Reagents

Copper sulfate pentahydrate (CuSO_4_·5H_2_O, catalog number: 6089) purchased from Duksan Pure Chemicals Co., Ltd. (Ansan, Republic of Korea) was used as the copper source in all experiments. Stock solutions were filter-sterilized and added to autoclaved YPD medium to prepare copper-supplemented media with final concentrations ranging from 0.25 to 2.5 g/L.

### 4.3. Determination of the Growth Inhibitory Concentration of Copper

To determine the concentration at which copper inhibited yeast growth, *S. cerevisiae* W303 was inoculated into 20 mL of YPD medium containing varying concentrations of CuSO_4_·5H_2_O (0.25 to 2.5 g/L) in 250 mL Erlenmeyer baffled culture flasks. The cells were cultured at 30 °C and 250 rpm for 20 h. The cell density was measured in terms of CFUs. The culture was serially diluted, and 100 μL of each dilution was spread onto YPD agar plates (YPD medium with 1.5% agar). The plates were incubated at 30 °C, and the number of CFUs was counted.

### 4.4. Development of Copper-Tolerant Mutants

Cells were inoculated at Abs_600_ (absorbance at 600 nm) = 0.1 and serially subcultured (1:100 dilution) every 24 h. Copper-resistant strains were generated using adaptive evolution. Wild-type *S. cerevisiae* W303 was initially cultured in 1.75 g/L copper-supplemented YPD medium, a concentration known to partially inhibit growth. Cells were serially subcultured every 24 h under the same copper concentration until their growth rate approximated that of the wild-type population in 1.5 g/L CuSO_4_·5H_2_O. Subsequently, the adapted population was subjected to further serial subculturing in 2.5 g/L CuSO_4_·5H_2_O to develop full resistance. To confirm that the obtained copper-resistant mutant was *S. cerevisiae*, a partial 18S ribosomal RNA gene sequence was analyzed by Macrogen Inc. (Seoul, Republic of Korea). The resulting sequence was then subjected to a BLAST search in the NCBI database, which confirmed its identity as *S. cerevisiae*.

### 4.5. Growth Comparison Between Wild-Type and Mutant Strains

To compare the growth performance of the wild-type and copper-resistant mutant strains, each strain was cultured in both standard YPD and copper-supplemented (2.5 g/L CuSO_4_·5H_2_O) YPD medium. Cells were inoculated into 5 mL of YPD medium for pre-culturing, followed by incubation at 30 °C and 250 rpm for 18 h. Main cultures were inoculated with an initial Abs_600_ = 0.1 and grown in 100 mL of YPD medium or YPD medium supplemented with 2.5 g/L CuSO_4_·5H_2_O in 500 mL Erlenmeyer baffled culture flasks at 30 °C and 250 rpm. Abs_600_ was also used to measure cell density every 2 h using a UV spectrophotometer (Optizen 2120UV; Mecasys Co., Ltd., Daejeon, Republic of Korea).

### 4.6. Total RNA Extraction

*Saccharomyces cerevisiae* W303 and the copper-resistant mutant strain (WT and MT, respectively) were inoculated at Abs_600_ = 0.1 in 99 mL of YPD medium at 30 °C and 250 rpm. After 8 h of incubation, 1 mL of either sterile water (for the control condition) or 1 mL of 0.25 g/mL CuSO_4_·5H_2_O solution (for the copper-stress condition) was added to reach a final copper concentration of 2.5 g/L. Cultures were continued until 14 h of growth, corresponding to the late exponential phase just before stationary phase. Samples cultured in standard YPD medium were labeled “NO”, while those exposed to 2.5 g/L CuSO_4_·5H_2_O were labeled “CU”. Thus, WT_NO and MT_CU refer to the wild-type and mutant strains under non-stress and copper-stressed conditions, respectively. The final numeric digit indicated the batch or biological replicate, and CU samples without a digit represented the average of CU1 and CU2.

Cultures were harvested at late-log phase and washed twice with 0.01 M EDTA before RNA extraction to eliminate extracellular Cu^2+^. Total RNA was extracted using Trizol reagent (catalog number: 15596018; Thermo Fisher Scientific Korea Ltd., Seoul, Republic of Korea). To 0.25 mL of the previously collected sample, 0.75 mL of the Trizol reagent was added and incubated at room temperature for 5 min. After this, 0.15 mL of chloroform was added, followed by vigorous shaking for 15 s and incubation at room temperature for a further 3 min. The mixture was centrifuged at 12,000× *g* for 15 min at 4 °C, and the upper aqueous phase was transferred to a new tube. To this, 0.375 mL of isopropyl alcohol was added and incubated for 10 min at room temperature. After incubation, the sample was centrifuged again at 12,000× *g* for 10 min at 4 °C, and the supernatant was removed, leaving only the RNA pellet. To wash the pellet, 0.75 mL of 75% ethanol was added to the tube and gently vortexed. The mixture was then centrifuged at 7500× *g* for 5 min at 4 °C, and the ethanol was discarded. The pellet was air-dried at room temperature for 10 min. The final RNA pellet was resuspended in RNase-free water. The resuspended RNA was evaluated using gel electrophoresis to assess the size distribution of the RNA fragments and the extent of RNA degradation.

### 4.7. RNA Quality Control

RNA integrity and concentration were assessed by Macrogen Inc. (Seoul, Republic of Korea) using a 2100 Bioanalyzer (Agilent Technologies, Inc., Santa Clara, CA, USA). Samples with suitable RNA integrity and purity were selected for downstream microarray analysis.

### 4.8. Microarray Analysis

Gene expression profiling was conducted using an Affymetrix GeneChip Yeast Genome 2.0 Array platform (catalog number: 900554; Thermo Fisher Scientific Korea Ltd., Seoul, Republic of Korea). Microarray experiments were outsourced and conducted by Macrogen Inc. (Seoul, Republic of Korea). cDNA synthesis was performed using a GeneChip 3′ IVT Plus Reagent Kit (catalog number: 902416; Thermo Fisher Scientific Korea Ltd., Seoul, Republic of Korea) according to the manufacturer’s protocol. Biotin-labeled amplified RNA was synthesized from 100 ng of total RNA and subsequently fragmented via heat and ion-mediated hydrolysis at 94 °C for 35 min. A total of 12 μg of fragmented amplified RNA was hybridized to the microarray chip at 45 °C for 16 h in a hybridization oven.

After hybridization, the arrays were washed and stained using the GeneChip Fluidics Station 450 (catalog number: 00-0079; Thermo Fisher Scientific Korea Ltd., Seoul, Republic of Korea) and scanned on a GeneChip System 3000 (catalog number: 00-0218; Thermo Fisher Scientific Korea Ltd., Seoul, Republic of Korea). Raw data were processed using Affymetrix GeneChip Command Console software ver. 3.1.4 (Thermo Fisher Scientific Korea Ltd., Seoul, Republic of Korea) and Affymetrix Expression Console software R 3.0.2 (Thermo Fisher Scientific Korea Ltd., Seoul, Republic of Korea), which included background correction, summarization, and quantile normalization using the robust multiarray average (RMA) method. Two biological replicates were initially prepared per condition. RNA integrity was evaluated, and the samples with the highest RIN values were selected (one WT_NO2, one MT_NO2, and two MT_CU replicates). Pearson correlation coefficients among samples exceeded 0.986 (Figure 5), confirming the reproducibility of gene expression profiles.

### 4.9. Microarray Data Analysis

Microarray data were processed using Affymetrix GeneChip Command Console software (version 3.1, Affymetrix, Thermo Fisher Scientific Korea Ltd., Seoul, Republic of Korea) to generate CEL files. Raw signal intensities were background-corrected, normalized, and summarized using the RMA method implemented in Affymetrix^®^ Expression Console^TM^ software R 3.0.2.

DEGs were identified based on fold change thresholds of ≥2 or ≤−2 across eight pairwise comparisons among four samples (WT_NO2, MT_NO2, MT_CU1, and MT_CU2). The pairwise comparisons included MT_NO2 vs. WT_NO2, MT_CU1 vs. MT_NO2, MT_CU2 vs. MT_NO2, MT_CU1 vs. WT_NO2, MT_CU2 vs. WT_NO2, MT_CU2 vs. MT_CU1, MT_CU (average of CU1 and CU2) vs. MT_NO2, and MT_CU vs. WT_NO2. DEGs were identified using the Limma package in R, and statistical significance was assessed using the Benjamini–Hochberg method to adjust for the false discovery rate (FDR). Genes with an adjusted *p*-value < 0.05 were considered statistically significant. Hierarchical clustering (Euclidean distance, complete linkage) and multidimensional scaling were employed to visualize transcriptomic similarities and differences between samples.

To evaluate their functional relevance, the DEGs were functionally annotated and clustered using DAVID Bioinformatics Resources (version 6.8, Leidos Biomedical Research, Inc.). Enrichment analysis included Gene Ontology (GO) terms, Kyoto Encyclopedia of Genes and Genomes pathways, and UniProt keywords. Annotation clusters with an enrichment score of ≥1.3 (*p* < 0.05) were considered significant. Genes within each cluster were analyzed to identify associations with copper resistance mechanisms, including metal ion transport, oxidative stress responses, and protein folding.

Hierarchical clustering was visualized using the heatmap.2 function in the gplots package, and multidimensional scaling plots were generated to illustrate the overall transcriptomic landscape. Clustered genes were further categorized based on their functional annotations to identify distinct expression patterns related to copper stress adaptation.

## 5. Conclusions

This study demonstrates that adaptive evolution and transcriptomic profiling can effectively elucidate the genetic mechanisms underlying copper resistance in *S. cerevisiae*. The identified copper-resistant mutant strain exhibited robust growth under copper stress conditions, accompanied by the upregulation of key genes involved in copper transport, oxidative stress response, and metal ion detoxification. Specifically, the overexpression of *ATX1*, *CTR1*, and *FRE3* highlights the significance of copper homeostasis and controlled metal uptake in mitigating copper-induced toxicity. Additionally, the upregulation of *RCK1* and *SOD1* suggests that oxidative stress response pathways protect against copper-generated ROS. The findings also identify SLF1 as a potential regulator of cellular metal ion balance under copper stress, while the observed changes in the RNA integrity of the copper-resistant mutant, characterized by RNA fragmentation and altered rRNA profiles, suggest that copper stress not only affects gene expression but also induces transcriptomic reprogramming involving RNA processing pathways.

These results provide useful insights into the molecular architecture of copper resistance in eukaryotic microorganisms. In particular, the genes identified as being associated with copper resistance represent potential molecular targets for the development of enhanced, copper-based wood preservatives designed to combat copper-tolerant fungal pathogens. By targeting pathways involved in metal ion transport, oxidative stress mitigation, and cellular detoxification, novel preservative formulations could be developed to prolong the effectiveness of copper treatments for wood, thus reducing fungal colonization and suppressing wood degradation. Future studies should explore the functional roles of newly identified stress-responsive genes under varying environmental conditions to facilitate the development of more sustainable and durable wood preservation strategies.

## Figures and Tables

**Table 1:** Candidate genes involved in copper resistance in *S. cerevisiae*. The fold change was evaluated by comparing the level of mRNA from the copper resistance mutant under copper stress with the level of mRNA from the wild-type strain without copper stress.

Gene	Systematic Name	Fold Change	Function	Copper-Related Function	References
*ATX1*	YNL259C	4.48	Cytosolic copper metallochaperone	Intracellular copper transport & detoxification	[22]
*CTR1*	YPR124W	−3.60	High-affinity copper uptake transporter	Downregulated to reduce copper influx	[23]
*FRE3*	YOR381W	2.05	Ferric reductase	Copper- and iron-responsive membrane protein	[24]
*SLF1*	YDR515W	2.32	Copper mineralization and RNA binding protein	Detoxifies extracellular copper by CuS precipitation	[25,26]

**Table 2:** Stress-related genes upregulated more than two-fold in copper-exposed mutant strains. The fold change was evaluated by comparing the mRNA levels from the copper-resistant mutant under copper stress with the mRNA levels from the wild-type strain without copper stress.

Gene	Systematic Name	Fold Change	Function	Copper-Related Function	References
*CDC37*	YDR168W	2.68	Hsp90 co-chaperone; protein folding	Protein folding under stress	[27,28]
*COX5B*	YIL111W	2.04	Mitochondrial cytochrome c oxidase subunit	Mitochondrial adaptation to stress	[29]
*GIM4*	YEL003W	3.08	Tubulin-binding cytosolic chaperonin	Cytoskeleton stability under stress	[30,31]
*JJJ2*	YJL162C	2.73	Hsp40-type co-chaperone	Protein homeostasis in stress	[32]
*PFD1*	YJL179W	3.42	Prefoldin subunit; assists in protein folding	Proteostasis under metal stress	[33]
*RCK1*	YGL158W	4.02	Oxidative stress-responsive kinase	Signal transduction in ROS response	[34]
*SNO4*	YMR322C	2.99	Stress-responsive peptidase-like protein	Oxidative or environmental stress	[35]
*SSA4*	YER103W	2.42	Hsp70 family heat shock protein	Heat and protein misfolding stress	[36]

## Data Availability

The original contributions presented in this study are included in the article. Further inquiries can be directed at the corresponding author.

## Supplementary Materials

The following supporting information can be downloaded at: https://www.mdpi.com/article/10.3390/antibiotics14111152/s1, Figure S1: The small subunit ribosomal RNA gene sequence of the copper-resistant mutant; Figure S2: Total RNA analysis using gel electrophoresis; Figure S3: Boxplot of normalized signal intensities across microarray samples; Figure S4: Density plot of normalized microarray signal distributions across all samples; Figure S5: Control probe performance for the microarray samples; Figure S6: Hierarchical clustering of gene expression profiles across all samples; Figure S7: Multidimensional scaling plot of gene expression profiles for the microarray samples; Figure S8: Heatmap and hierarchical clustering of differentially expressed genes (DEGs) for all samples; Table S1: Top 10 matching sequences showed the highest similarity to the ribosomal RNA gene of the copper-resistant Saccharomyces cerevisiae mutant; Table S2: Differential gene expression analysis: Relative expression ratios (fold changes) under experimental conditions.

## Abbreviations

The following abbreviations are used in this manuscript:

Abs_600_	Absorbance at 600 nm
ALE	Adaptive laboratory evolution
CFUs	Colony-forming units
DEGs	Differentially expressed genes
GO	Gene ontology
MIC	Minimal inhibitory concentration
ROS	Reactive oxygen species
