# Supplementary material for: Transcriptomic Analysis of Copper Resistance in Saccharomyces cerevisiae: Insights into Adaptive Evolution and Potential Implications for Wood Preservative Treatments
Source: Antibiotics (Basel). 2025 Nov 14;14(11):1152. doi: 10.3390/antibiotics14111152 (PMC12649743; doi:10.3390/antibiotics14111152)
Supplement: Supplementary file 1 [file antibiotics-14-01152-s001.zip › Supplementary data 1.pptx]

## Slide 1
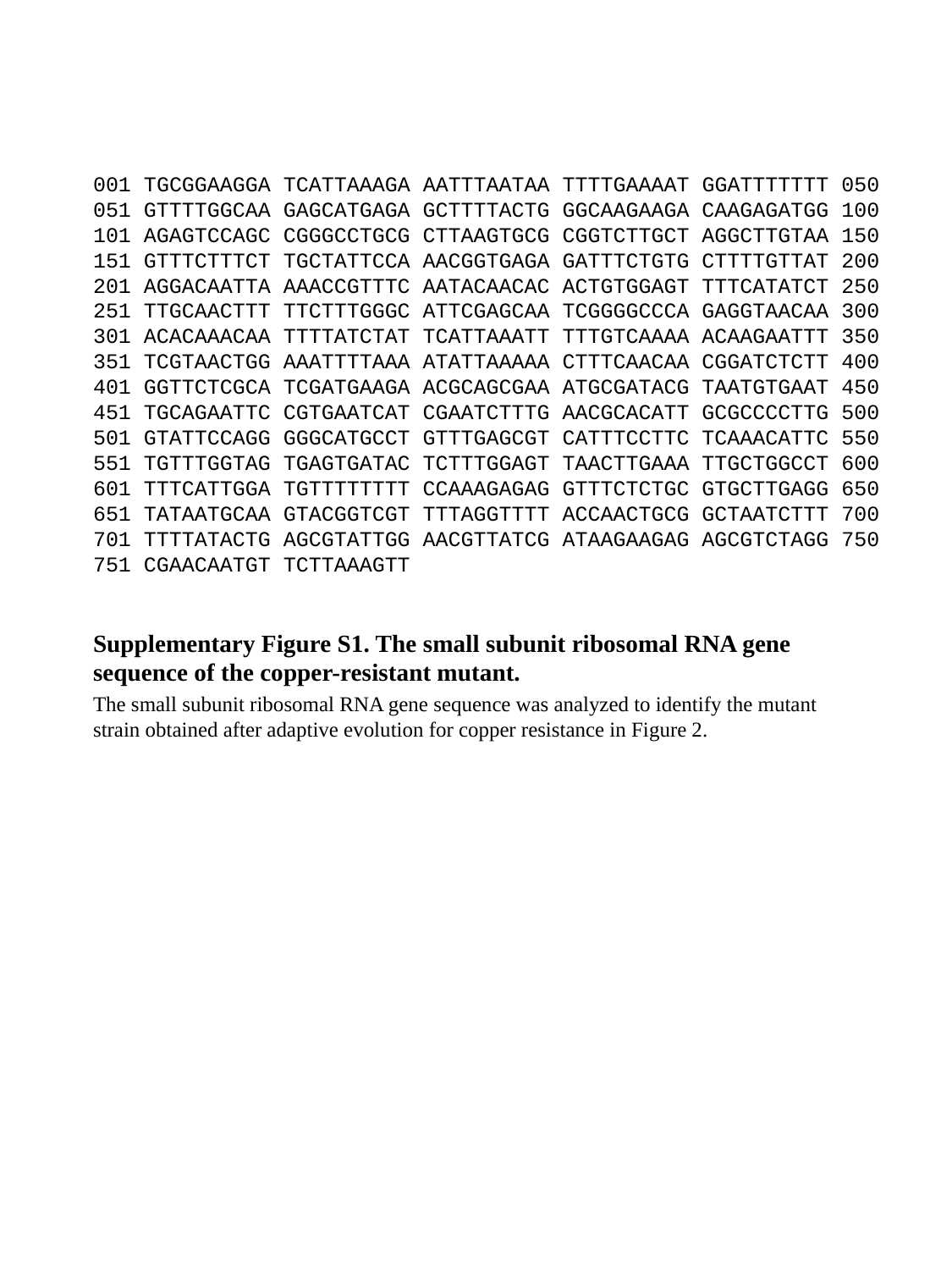

001 Tgcggaagga tcattaaaga aatttaataa ttttgaaaat ggattttttt 050 051 gttttggcaa gagcatgaga gcttttactg ggcaagaaga caagagatgg 100 101 agagtccagc cgggcctgcg cttaagtgcg cggtcttgct aggcttgtaa 150 151 gtttctttct tgctattcca aacggtgaga gatttctgtg cttttgttat 200 201 aggacaatta aaaccgtttc aatacaacac actgtggagt tttcatatct 250 251 ttgcaacttt ttctttgggc attcgagcaa tcggggccca gaggtaacaa 300 301 acacaaacaa ttttatctat tcattaaatt tttgtcaaaa acaagaattt 350 351 tcgtaactgg aaattttaaa atattaaaaa ctttcaacaa cggatctctt 400 401 ggttctcgca tcgatgaaga acgcagcgaa atgcgatacg taatgtgaat 450 451 tgcagaattc cgtgaatcat cgaatctttg aacgcacatt gcgccccttg 500 501 gtattccagg gggcatgcct gtttgagcgt catttccttc tcaaacattc 550 551 tgtttggtag tgagtgatac tctttggagt taacttgaaa ttgctggcct 600 601 tttcattgga tgtttttttt ccaaagagag gtttctctgc gtgcttgagg 650 651 tataatgcaa gtacggtcgt tttaggtttt accaactgcg gctaatcttt 700 701 ttttatactg agcgtattgg aacgttatcg ataagaagag agcgtctagg 750 751 cgaacaatgt tcttaaagtt
Supplementary Figure S1. The small subunit ribosomal RNA gene sequence of the copper-resistant mutant.
The small subunit ribosomal RNA gene sequence was analyzed to identify the mutant strain obtained after adaptive evolution for copper resistance in Figure 2.

## Slide 2
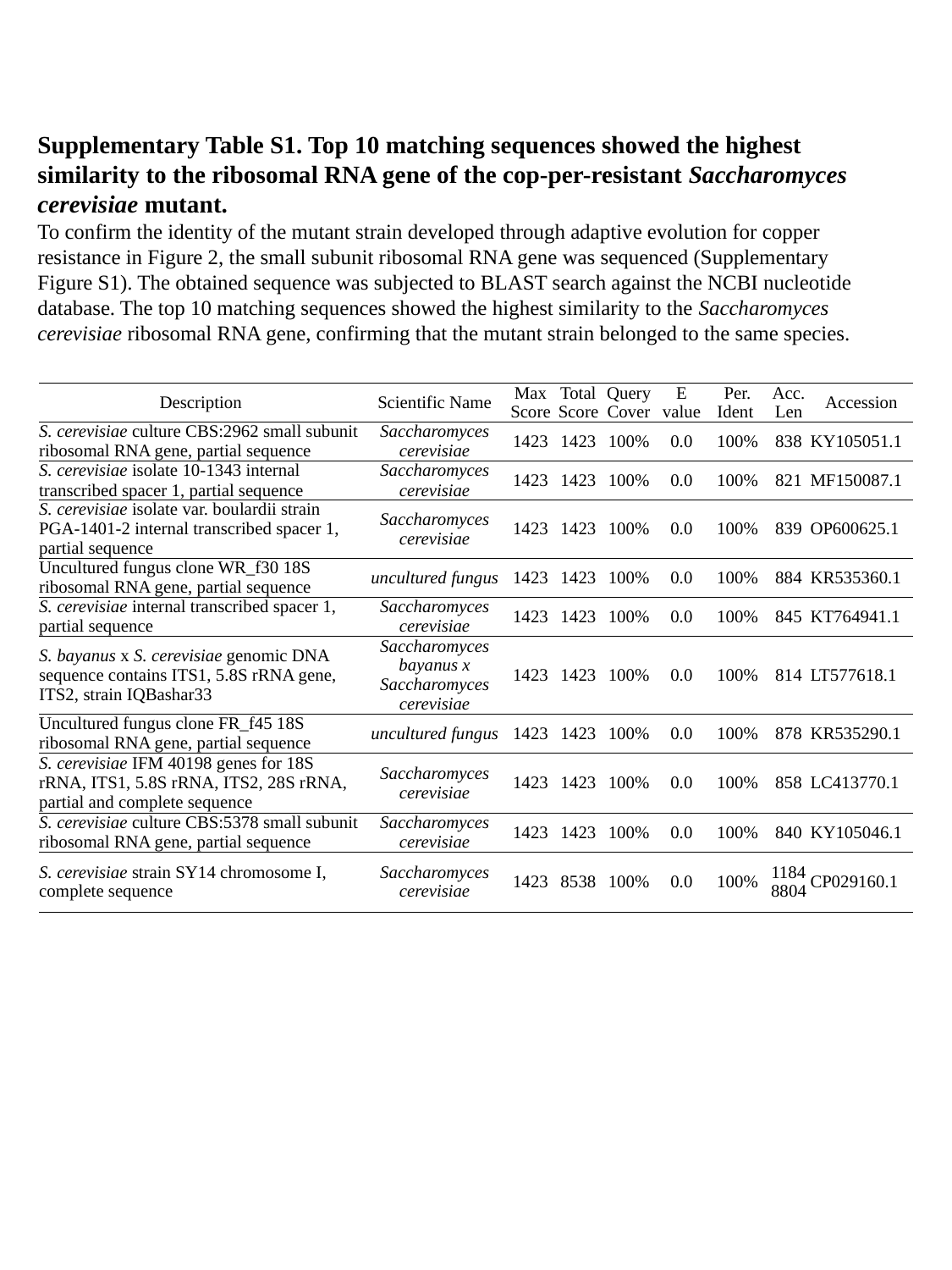

Supplementary Table S1. Top 10 matching sequences showed the highest similarity to the ribosomal RNA gene of the cop-per-resistant Saccharomyces cerevisiae mutant.
To confirm the identity of the mutant strain developed through adaptive evolution for copper resistance in Figure 2, the small subunit ribosomal RNA gene was sequenced (Supplementary Figure S1). The obtained sequence was subjected to BLAST search against the NCBI nucleotide database. The top 10 matching sequences showed the highest similarity to the Saccharomyces cerevisiae ribosomal RNA gene, confirming that the mutant strain belonged to the same species.
| Description | Scientific Name | Max Score | Total Score | Query Cover | E value | Per. Ident | Acc. Len | Accession |
| --- | --- | --- | --- | --- | --- | --- | --- | --- |
| S. cerevisiae culture CBS:2962 small subunit ribosomal RNA gene, partial sequence | Saccharomyces cerevisiae | 1423 | 1423 | 100% | 0.0 | 100% | 838 | KY105051.1 |
| S. cerevisiae isolate 10-1343 internal transcribed spacer 1, partial sequence | Saccharomyces cerevisiae | 1423 | 1423 | 100% | 0.0 | 100% | 821 | MF150087.1 |
| S. cerevisiae isolate var. boulardii strain PGA-1401-2 internal transcribed spacer 1, partial sequence | Saccharomyces cerevisiae | 1423 | 1423 | 100% | 0.0 | 100% | 839 | OP600625.1 |
| Uncultured fungus clone WR\_f30 18S ribosomal RNA gene, partial sequence | uncultured fungus | 1423 | 1423 | 100% | 0.0 | 100% | 884 | KR535360.1 |
| S. cerevisiae internal transcribed spacer 1, partial sequence | Saccharomyces cerevisiae | 1423 | 1423 | 100% | 0.0 | 100% | 845 | KT764941.1 |
| S. bayanus x S. cerevisiae genomic DNA sequence contains ITS1, 5.8S rRNA gene, ITS2, strain IQBashar33 | Saccharomyces bayanus x Saccharomyces cerevisiae | 1423 | 1423 | 100% | 0.0 | 100% | 814 | LT577618.1 |
| Uncultured fungus clone FR\_f45 18S ribosomal RNA gene, partial sequence | uncultured fungus | 1423 | 1423 | 100% | 0.0 | 100% | 878 | KR535290.1 |
| S. cerevisiae IFM 40198 genes for 18S rRNA, ITS1, 5.8S rRNA, ITS2, 28S rRNA, partial and complete sequence | Saccharomyces cerevisiae | 1423 | 1423 | 100% | 0.0 | 100% | 858 | LC413770.1 |
| S. cerevisiae culture CBS:5378 small subunit ribosomal RNA gene, partial sequence | Saccharomyces cerevisiae | 1423 | 1423 | 100% | 0.0 | 100% | 840 | KY105046.1 |
| S. cerevisiae strain SY14 chromosome I, complete sequence | Saccharomyces cerevisiae | 1423 | 8538 | 100% | 0.0 | 100% | 11848804 | CP029160.1 |

## Slide 3
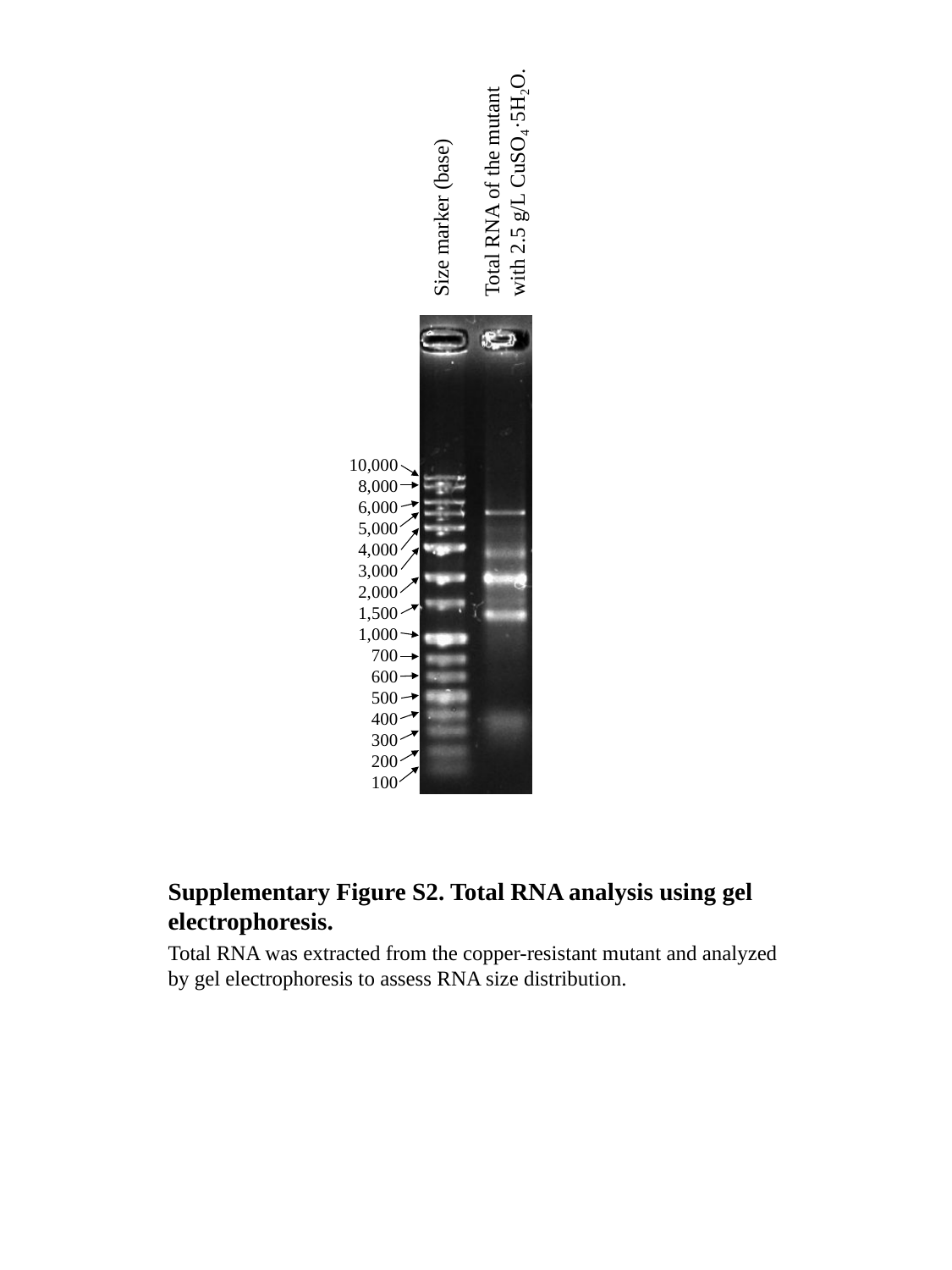

Size marker (base)
Total RNA of the mutant
with 2.5 g/L CuSO₄·5H₂O.
10,000
8,000
6,000
5,000
4,000
3,000
2,000
1,500
1,000
700
600
500
400
300
200
100
Supplementary Figure S2. Total RNA analysis using gel electrophoresis.
Total RNA was extracted from the copper-resistant mutant and analyzed by gel electrophoresis to assess RNA size distribution.

## Slide 4
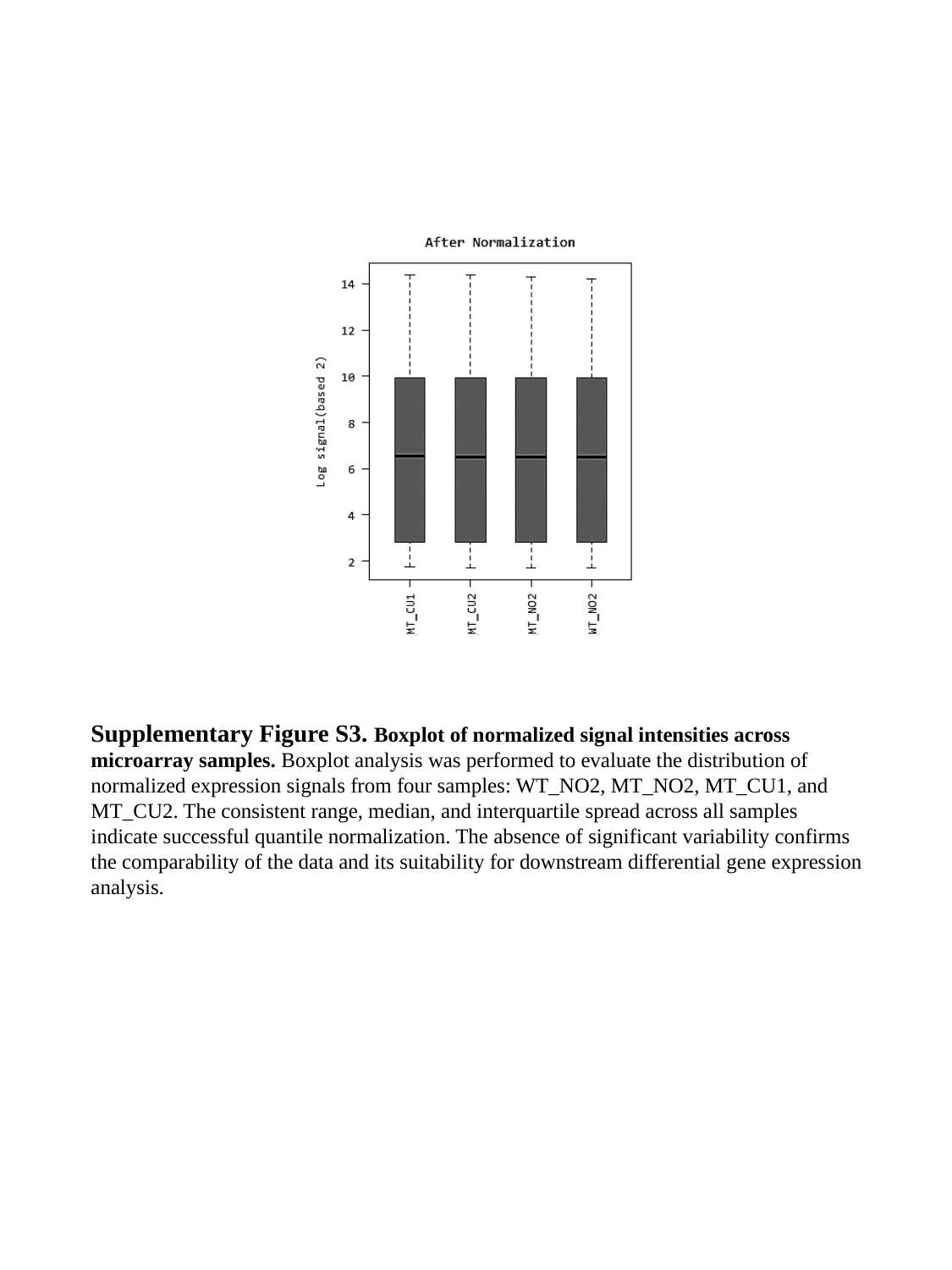

Supplementary Figure S3. Boxplot of normalized signal intensities across microarray samples. Boxplot analysis was performed to evaluate the distribution of normalized expression signals from four samples: WT_NO2, MT_NO2, MT_CU1, and MT_CU2. The consistent range, median, and interquartile spread across all samples indicate successful quantile normalization. The absence of significant variability confirms the comparability of the data and its suitability for downstream differential gene expression analysis.

## Slide 5
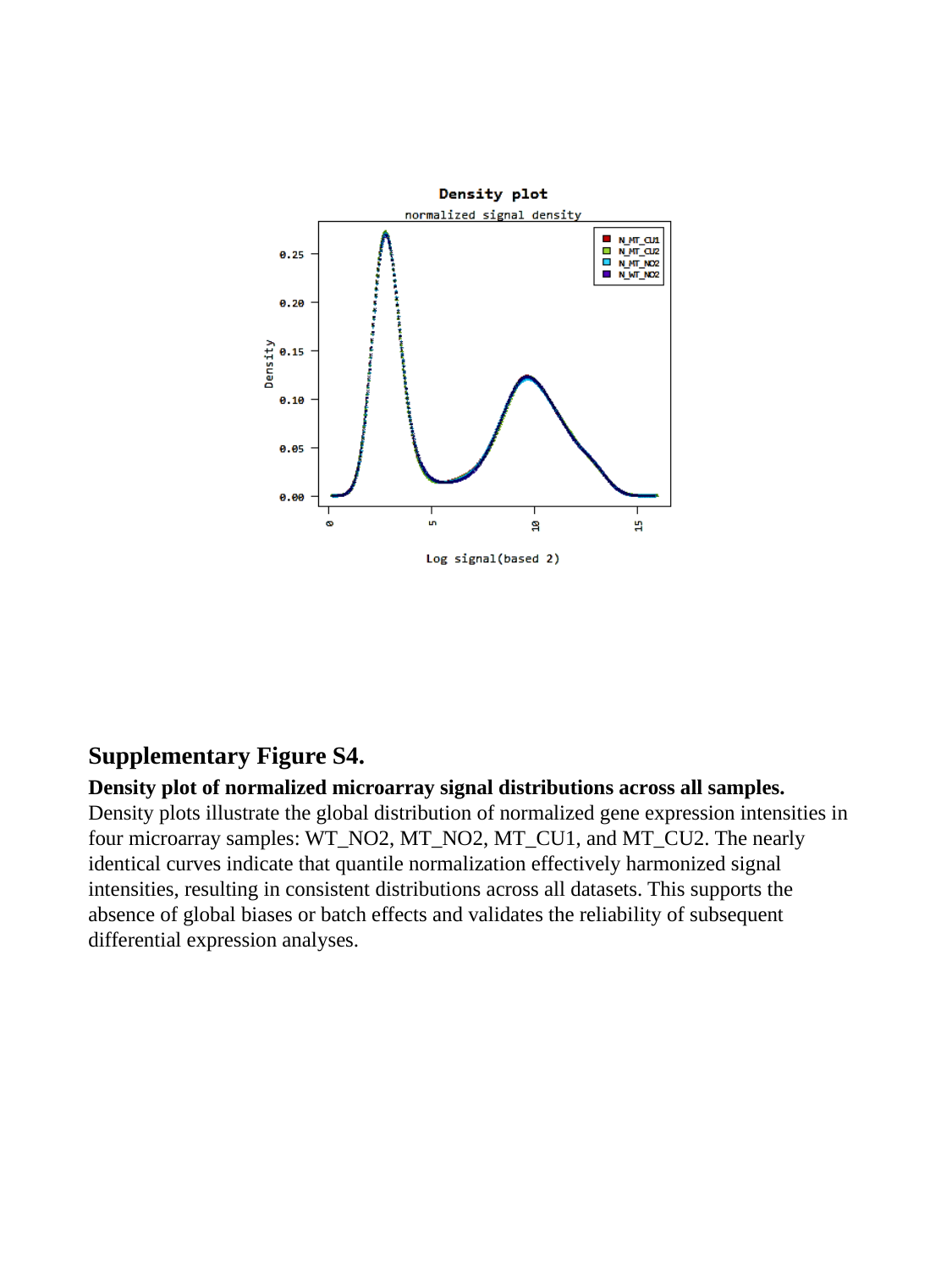

Supplementary Figure S4.
Density plot of normalized microarray signal distributions across all samples.
Density plots illustrate the global distribution of normalized gene expression intensities in four microarray samples: WT_NO2, MT_NO2, MT_CU1, and MT_CU2. The nearly identical curves indicate that quantile normalization effectively harmonized signal intensities, resulting in consistent distributions across all datasets. This supports the absence of global biases or batch effects and validates the reliability of subsequent differential expression analyses.

## Slide 6
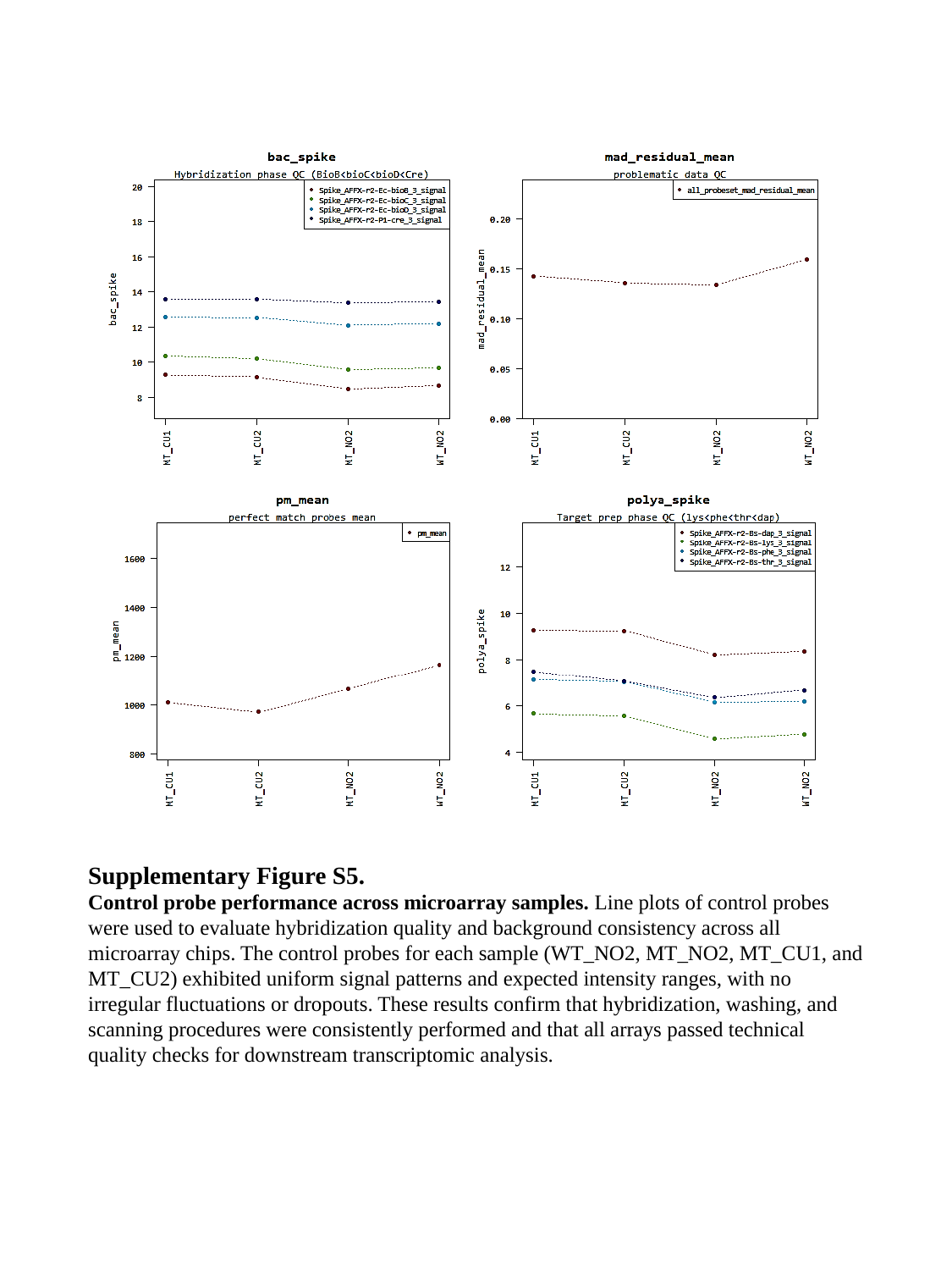

Supplementary Figure S5.
Control probe performance across microarray samples. Line plots of control probes were used to evaluate hybridization quality and background consistency across all microarray chips. The control probes for each sample (WT_NO2, MT_NO2, MT_CU1, and MT_CU2) exhibited uniform signal patterns and expected intensity ranges, with no irregular fluctuations or dropouts. These results confirm that hybridization, washing, and scanning procedures were consistently performed and that all arrays passed technical quality checks for downstream transcriptomic analysis.

## Slide 7
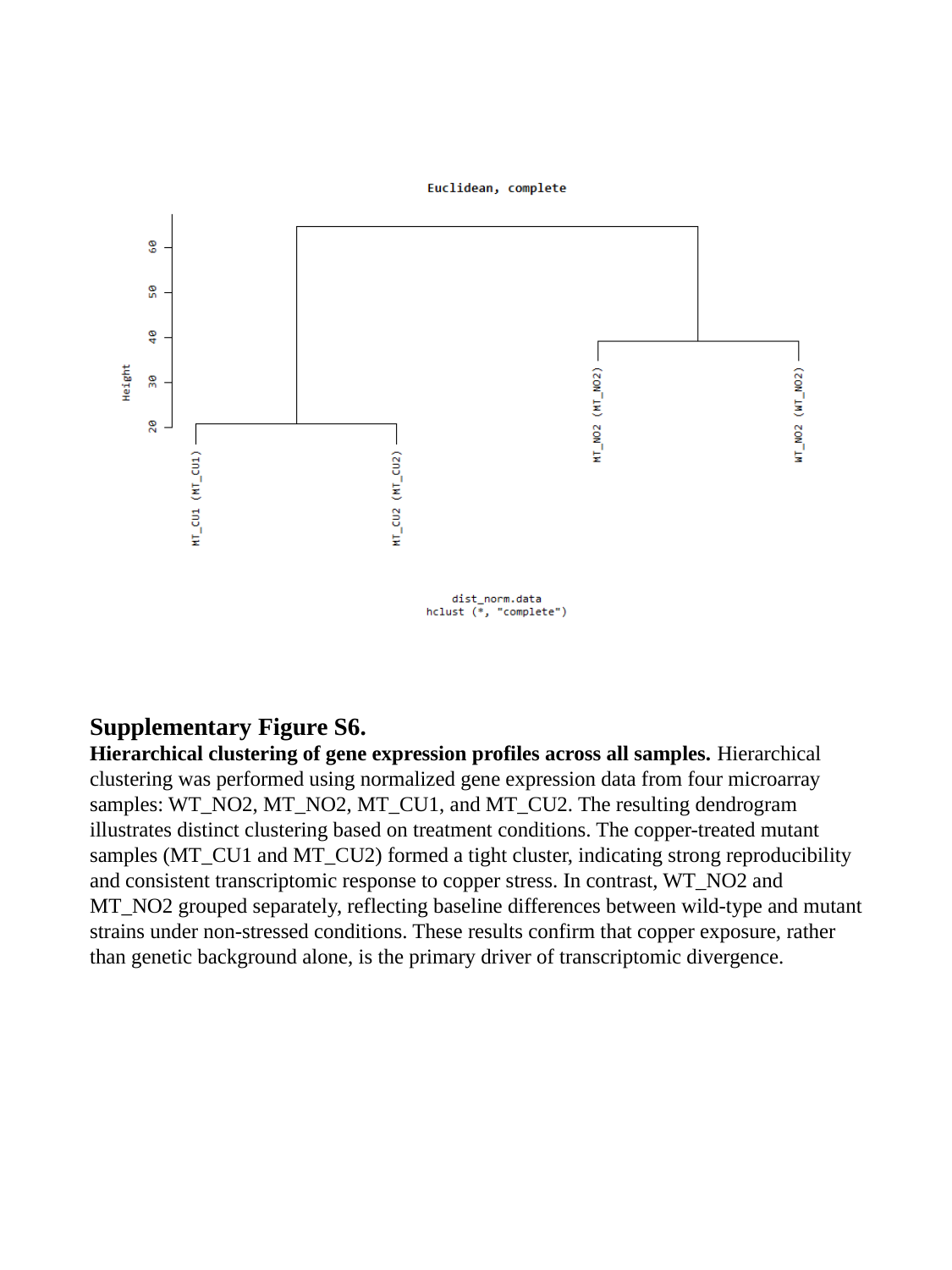

Supplementary Figure S6.
Hierarchical clustering of gene expression profiles across all samples. Hierarchical clustering was performed using normalized gene expression data from four microarray samples: WT_NO2, MT_NO2, MT_CU1, and MT_CU2. The resulting dendrogram illustrates distinct clustering based on treatment conditions. The copper-treated mutant samples (MT_CU1 and MT_CU2) formed a tight cluster, indicating strong reproducibility and consistent transcriptomic response to copper stress. In contrast, WT_NO2 and MT_NO2 grouped separately, reflecting baseline differences between wild-type and mutant strains under non-stressed conditions. These results confirm that copper exposure, rather than genetic background alone, is the primary driver of transcriptomic divergence.

## Slide 8
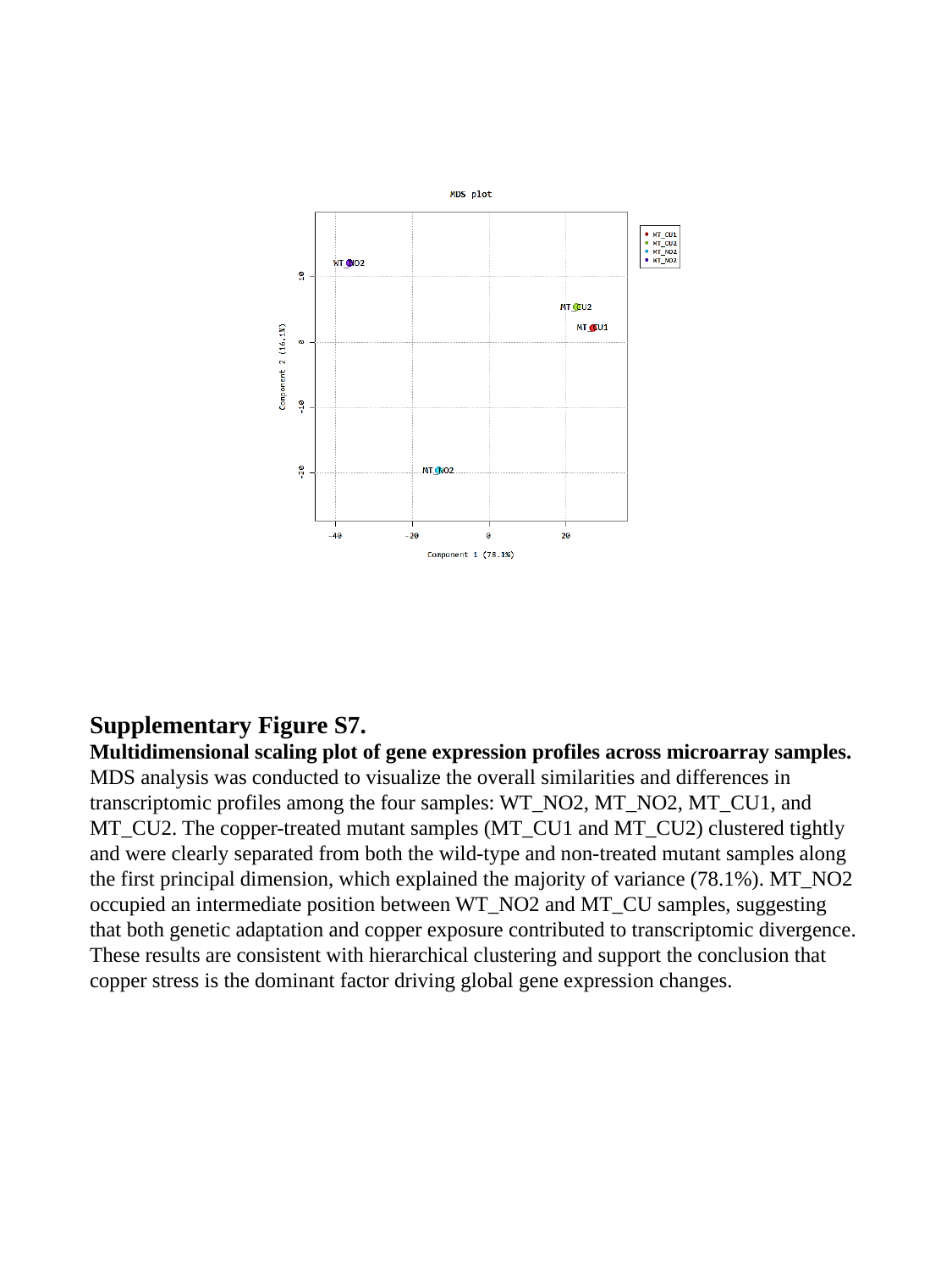

Supplementary Figure S7.
Multidimensional scaling plot of gene expression profiles across microarray samples. MDS analysis was conducted to visualize the overall similarities and differences in transcriptomic profiles among the four samples: WT_NO2, MT_NO2, MT_CU1, and MT_CU2. The copper-treated mutant samples (MT_CU1 and MT_CU2) clustered tightly and were clearly separated from both the wild-type and non-treated mutant samples along the first principal dimension, which explained the majority of variance (78.1%). MT_NO2 occupied an intermediate position between WT_NO2 and MT_CU samples, suggesting that both genetic adaptation and copper exposure contributed to transcriptomic divergence. These results are consistent with hierarchical clustering and support the conclusion that copper stress is the dominant factor driving global gene expression changes.

## Slide 9
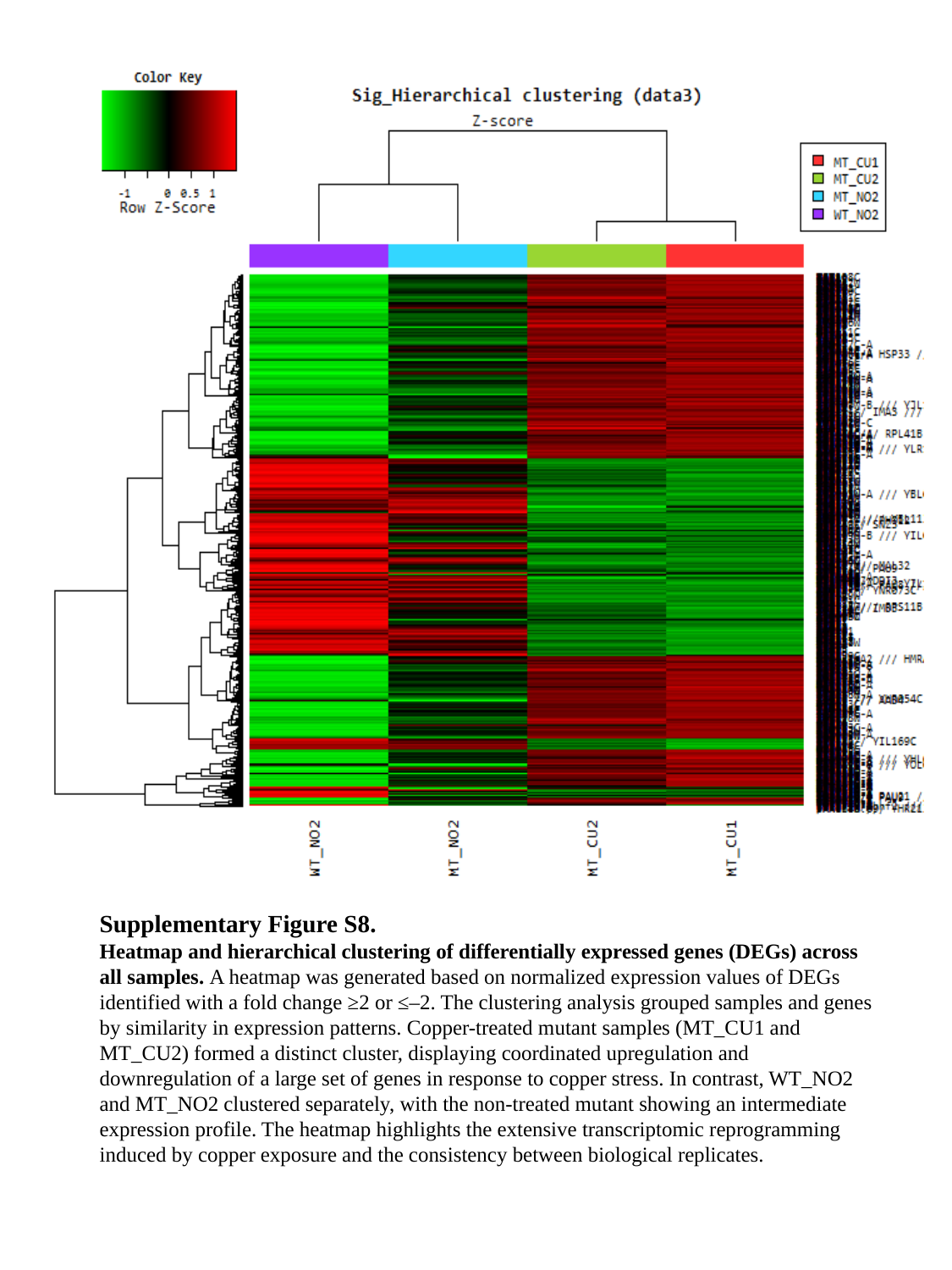

Supplementary Figure S8.
Heatmap and hierarchical clustering of differentially expressed genes (DEGs) across all samples. A heatmap was generated based on normalized expression values of DEGs identified with a fold change ≥2 or ≤–2. The clustering analysis grouped samples and genes by similarity in expression patterns. Copper-treated mutant samples (MT_CU1 and MT_CU2) formed a distinct cluster, displaying coordinated upregulation and downregulation of a large set of genes in response to copper stress. In contrast, WT_NO2 and MT_NO2 clustered separately, with the non-treated mutant showing an intermediate expression profile. The heatmap highlights the extensive transcriptomic reprogramming induced by copper exposure and the consistency between biological replicates.
